# TDP-43 dysregulation impairs cholesterol metabolism linked with myelination defects

**DOI:** 10.1007/s00401-025-02927-x

**Published:** 2025-09-04

**Authors:** Irene García-Toledo, Juan M. Godoy-Corchuelo, Luis C. Fernández-Beltrán, Zeinab Ali, Ariadna Guindo-Arroyo, Irene Jiménez-Coca, Jesús Jiménez-Rodríguez, Karen Javaloyes-García, Marcos Viñuela, Ulises Gómez-Pinedo, Laura Saiz-Aúz, Alberto Rábano, Estela Área-Gómez, Thomas J. Cunningham, Silvia Corrochano

**Affiliations:** 1 Neurological Disorders Group, Hospital Clínico San Carlos, IdISSC, Madrid, Spain; 2 Department of Medicine, Universidad Complutense de Madrid, Madrid, Spain; 3 Inmunotek S.L., Alcalá de Henares, Madrid, Spain; 4 Department of Immunology, Hospital Clínico San Carlos, IdISSC, Madrid, Spain; 5 Reina Sofía Alzheimer Center, CIEN Foundation, ISCIII, Madrid, Spain; 6 Centro de Investigaciones Biológicas “Margarita Salas”, Madrid, Spain; 7 MRC Prion Unit at UCL, UCL Institute of Prion Diseases, London, UK

**Keywords:** TDP-43 proteinopathies, Cholesterol metabolism, Lipidomics, Transcriptomics, Myelin, Lipid droplets

## Abstract

TDP-43 is a nuclear protein encoded by the *TARDBP* gene, which forms pathological aggregates in various neurodegenerative diseases, collectively known as TDP-43 proteinopathies, including amyotrophic lateral sclerosis (ALS) and frontotemporal dementia (FTD). These diseases are characterized by multiple pathological mechanisms, with disruptions in lipid regulatory pathways emerging as a critical factor. However, the role of TDP-43 in the regulation of the brain lipid homeostasis and the potential connection of TDP-43 dysfunction to myelin alterations in TDP-43 proteionopathies remain poorly understood, despite the fact that lipids, particularly cholesterol, comprise nearly 70% of myelin. To investigate the causal relationship between TDP-43 dysfunction and disruptions in brain cholesterol homeostasis, we conducted multi-omics analyses (lipidomics, transcriptomics, and functional splicing) on the frontal cortex from the *Tardbp*^*M323K/M323K*^ knock-in mouse model. Lipidomic analysis revealed alterations in lipid pathways related to membrane composition and lipid droplet accumulation, particularly affecting cholesterol-related species. We found higher lipid droplet accumulation in primary fibroblasts derived from these mice, as well as in the brain of the mutant mice. Similarly, the immunohistochemical detection of a lipid droplet marker was higher in the postmortem frontal cortex, gray matter, and white matter of FTLD-TDP patients compared to non-neurological controls. Transcriptomic analyses showed that TDP-43 pathology led to transcriptional dysregulation of genes essential for myelin production and maintenance. We identified impaired cholesterol metabolism, mainly through the downregulation of endogenous cholesterol synthesis, alongside upregulated cholesterol transport pathways, which we further replicated in FTLD-TDP patients transcriptomic datasets. Collectively, our findings suggest that TDP-43 dysfunction disrupts brain cholesterol homeostasis, potentially compromising myelin integrity.

**Supplementary Information:**

The online version contains supplementary material available at 10.1007/s00401-025-02927-x.

## Introduction

TDP-43 proteinopathies are a group of neurodegenerative disorders characterized by the mislocalization and aggregation of transactive response DNA-binding protein 43 (TDP-43) in the cytoplasm of neurons and glial cells. TDP-43, a highly conserved RNA/DNA-binding protein, plays a critical role in RNA metabolism, including transcriptional regulation, RNA splicing, transport, and stress granule formation [3, 43]. Its pathological accumulation leads to loss of nuclear function and toxic gain of function in the cytoplasm, contributing to neurodegeneration. TDP-43 proteinopathies are most commonly associated with amyotrophic lateral sclerosis (ALS) and frontotemporal dementia (FTD), where TDP-43-positive inclusions are found in affected brain regions such as the motor cortex, spinal cord, and frontal and temporal lobes. Classically, the degeneration of gray matter, atrophy or loss, has been primarily associated with these disorders. In recent years, more studies are showing abnormalities, even reduction, of white matter in TDP-43 proteinopathies, as in the case of ALS [9, 31, 37] and FTD [30, 32]. These abnormalities include axonal degeneration, myelin loss, and gliosis, further contributing to the cognitive and motor impairments observed.

Nearly 40% of the brain is white matter, which is highly packed fibers with myelin as the central component. The myelin sheets are lipid-rich membranes that isolate the axons of neurons essential for the correct axonal electrical conduction in the brain. The main lipids that make up the myelin are cholesterol, phospholipids, and glycolipids (galactocerebroside) in ratios of ≈ 4:4:2 [24]. It has been estimated that up to 70% of the brain cholesterol is associated with myelin [45]. Cholesterol tight balance regulation is, therefore, essential for the proper functioning of myelin and brain function. For this reason, normal cholesterol biosynthesis is necessary for the maintenance of oligodendrocytes and is associated with myelin gene expression in vitro and myelin biosynthesis [21, 29, 39].

In TDP-43 proteinopathies, significant alterations in cholesterol metabolism and lipid profiles have been observed in both human patients and animal models. Lipidomics analyses of the frontal cortex of frontotemporal lobar degeneration with TDP-43 pathology (FTLD-TDP) patients had shown a marked disruption of lipid homeostasis, in particular a reduction in key lipid species such as ether lipids and acylcarnitines, which are essential for maintaining myelin integrity and neuronal function [2, 13]. Reduction in phospholipids was accompanied by an increase in cholesterol ester (CE) and triglycerides in human frontal cortex as well as in neurons derived from *C9ORF72* iPSC patients [16].

In recent years, more studies are supporting the role of TDP-43 in cholesterol homeostasis. TDP-43 binds the mRNA of the sterol regulatory element-binding protein 2 (SREBP2), a key transcription factor that regulates cholesterol biosynthesis, in oligodendrocytes. Both alterations of TDP-43 levels (depletion and overexpression) lead to reduced SREBP2 and cholesterol transport LDLR expression and activity, resulting in reduced cholesterol production in the spinal cord of mice and patients [12, 19]. This is important because cholesterol is a vital component of neuronal membranes and myelin, and its disruption has been linked to axonal degeneration and cognitive impairment [7, 34]. In addition, oligodendrocytes showed increased susceptibility to oxidative stress due to altered iron and lipid metabolism in the presence of TDP-43 pathology, exacerbating white matter degeneration [5, 20]. Disrupted cholesterol homeostasis in tissues has been detected in the form of cholesterol ester (CE) in abnormal accumulation in lipid droplets inside the cells. Indeed, lipid droplet accumulations have been found in the spinal cord of SOD1^G93A^ mice [11], as well as in astrocytes and neurons of TDP-43 transgenic mice and iPSC-derived motor neurons from *C9orf72* ALS/FTD patients, further supporting the role of cholesterol dysregulation in TDP-43 proteinopathies [1, 26, 36, 44].

We have previously characterized a mouse model of TDP-43 proteinopathy, the *Tardbp*^*M323K/M323K*^ mice, which harbors a physiological point mutation in the C-terminal domain of the TDP-43 protein [15, 17]. This mutation recapitulates aspects of human neurodegenerative diseases associated with TDP-43 dysregulation. In this particular model, both cognitive and motor impairments manifest early in life and progressively worsen with age, providing a dynamic system to study the course and molecular mechanism of TDP-43-related alterations. From this model, we learned that TDP-43 is clearly crucial for correct brain development. The *Tardbp*^*M323K/M323K*^ mice have cognitive impairments, including learning and memory deficits with brain structural changes, particularly in regions associated with white matter integrity, including a reduction in the white matter volume, observed by structural MRI [15, 17].

Here, we investigated the role of TDP-43 in brain cholesterol homeostasis, which impacts on myelin integrity and in the correct neuronal functional activity. We used the *Tardbp*^*M323K/M323K*^ mice and FTLD-TDP patient samples to provide valuable insights into the relation of TDP-43 dysfunction and brain cholesterol dysregulation in TDP-43 proteinopathies.

## Methods

### Subjects studied

#### Human brain samples

Human brain samples were provided by Biobanks BT-CIEN and IdISSC Biobank (both in Madrid, Spain). Approved by the local ethical committee of Foundation CIEN (C:S20002). The control participants had no history of neurological disease, and their cause of death was unrelated to any neurological condition, including three women with an age range of 58–72 years and four men in the age range of 43–65. FTLD-TDP patients included two women with an age range of 80–84 years and five men in the age range of 54–75. FTLD-TAU patients included five patients with Pick disease and one with corticobasal degeneration.

TDP-43 pathology was assessed by histological staining using an antibody recognizing full-length TDP-43, and the criteria for pathological TDP-43 were the identification of cells with cytoplasmic TDP-43 inclusions without nuclear staining, and TDP-43-positive staining for fibers and neurites.

Frontal cortex co-pathology: our procedure for assessing Abeta, tau, and α-synuclein pathology allows us to predict with high probability what is happening in the frontal cortex. Only in cases with Thal stage > 0 would we expect to find Abeta in the frontal cortex. In none of the cases would we expect to find neurofibrillary tangles (NFT), Lewy bodies, or ARTAG lesions (there was no ARTAG in any of the cases) Table 1.

**Table 1:** Demographic and neuropathological data for the patient cohort

	Sex	Age	Pathological diagnosis	Brain area	Thal	ABC-A	ABC-C	Braak	ABC-B	Braak-a	LATE	HPC sclerosis	ARTAG	Vascular
1	Male	65	Control	N1 and CB	x	x	x	x	x	x	x	x	x	x
2	Male	64	Control	N1 and CB	x	x	x	x	x	x	x	x	x	x
3	Female	72	Control	N1 and CB	x	x	x	x	x	x	x	x	x	x
4	Male	58	Control	N1 and CB	x	x	x	x	x	x	x	x	x	x
5	Female	58	Control	FPC	0	0	0	0	0	0	0	0	0	1
6	Female	61	Control	FPC	1	1	0	1	1	0	0	0	0	0
7	Male	43	Control	FPC	0	0	0	0	0	0	0	0	0	0
8	Male	73	FTLD-TDP-C	FPC and CB	3	2	0	1	1	0	x	0	0	1
9	Female	84	FTLD-TDP-C	FPC and CB	0	0	0	2	1	0	x	0	0	0
10	Male	75	FTLD-TDP-B + C	FPC and CB	0	0	0	1	1	0	x	1	0	0
11	Male	68	FTLD-TDP-A	FPC and CB	0	0	0	1	1	1	x	1	0	0
12	Male	64	FTLD-TDP-B	FPC and CB	0	0	0	x	x	0	x	1	0	0
13	Female	80	FTLD-TDP-A	FPC and CB	2	1	0	2	1	0	x	1	0	1
14	Male	54	FTLD-TDP-B	FPC and CB	0	0	0	0	0	0	x	1	0	0
15	Male	59	FTLD-TAU	FPC	0	0	0	x	x	0	0	0	0	0
16	Male	64	FTLD-TAU	FPC	0	0	0	x	x	0	0	0	0	0
17	Female	75	FTLD-TAU	FPC	1	1	0	x	x	0	1	1	0	0
18	Female	86	FTLD-TAU	FPC	0	0	0	x	x	0	0	0	0	1
19	Male	65	FTLD-TAU	FPC	0	0	0	x	x	0	0	0	0	0
20	Female	79	FTLD-TAU	FPC	1	1	0	x	x	0	0	1	0	1

*FPC* fixed prefrontal cortex, *N1* N1 middle frontal gyrus, *FTLD-TDP-A* frontotemporal lobar degeneration with TDP-43 inclusions type A, *FTLD-TDP-B* frontotemporal lobar degeneration with TDP-43 inclusions type B, *FTLD-TDP-C* frontotemporal lobar degeneration with TDP-43 inclusions type C, *FTLD-TAU* frontotemporal lobar degeneration with Tau inclusions, *HPC sclerosis* hippocampal sclerosis, *X* no data, *0* absence, *1* presence; 1–3 in ThaI and Braak indicates the stage

#### Mice

The *Tardbp*^*M323K/M323K*^ mice were generated in a constant F1 DBA-C57BL hybrid background, as previously described [17]. For all the experiments, we use the wild-types (*Tardbp*^+*/*+^*)* and homozygous littermates (*Tardbp*^*M323K/M323K*^), and both sexes.

Mice were kept in auto-ventilated cages and fed “ad libitum” (Rat and Mouse Breeding 3 (RM3), Special Diet Services) with free access to water. Mice were kept under constant conditions with a regular 12 h light and dark cycle, a temperature of 21 ± 2 ^°^C, and humidity of 55 ± 10%. Mice were housed in same-sex cages of up to five mice. Cages contained Aspen Chips bedding (Datesand), shredded paper, and a rodent tunnel for enrichment. Separated male and female cohorts were used in most of the analyses unless specified. All mice were maintained and studied according to international, national, and regional guidelines. The study was approved by the local ethical committees of animal care and use of the Hospital Clínico San Carlos (Proex 258.4/20) in accordance with the European and Spanish regulations (2010/63/EU and RD 1201/2005).

At weaning, all mice were genotyped from the DNA extracted from an ear biopsy using the Phire Tissue Direct PCR Master Mix (Thermo Fisher Scientific) and the method of allele discrimination of Applied Biosystems, using a multiplexed qPCR assay (two primers and two probe pairs). The sequences of the primers and probes can be found at Supplementary Table 1.

### Techniques

#### Primary ear fibroblasts generation

The ears from wild-types (*Tardbp*^+*/*+^*)* and homozygous littermates (*Tardbp*^*M323K/M323K*^) (n = 5–6 mice, respectively) male mice at 3 months of age were removed and disinfected with 70% ethanol for 10 min, followed by three washes in PBS under a laminar flow hood. The ears were minced and placed in a sterile cryovial with 1–1.5 ml of a filtered enzyme solution containing collagenase D (Worthington) (2.5 mg/ml) and pre-activated pronase (Roche) (Pronase 20 mg/ml, Tris pH 8 10 mM, EDTA 1 mM), and activated at 37 °C for 30 min in complete RPMI medium (RPMI (Cultek), 10% FBS (Cultek), 2.5% HEPES (Thermo Fisher Scientific) and 1% antibiotic–antimycotic (Thermo Fisher Scientific)). The suspension was incubated for 95–100 min shaking (300 rpm) at 37 °C. After enzymatic digestion, the cells were disaggregated and passed through a 70 µm sieve into 20 ml of complete RPMI medium. The cell suspension was centrifuged at 500 g for 10 min and the pelleted fraction was resuspended in 10 ml of medium, filtered again and supplemented with a further 20 ml of medium before a second centrifugation. The final pelleted fraction was resuspended in 10–15 ml of complete RPMI medium and transferred to a cell culture flask for incubation. After 24 h, the medium was replaced with DMEM (Thermo Fisher Scientific) (DMEM high glucose, 10% FBS, 1% antibiotic–antimycotic), and the cells were cultured for 5 days, until they reached 80–90% confluency.

#### Proteins study

##### Immunohistochemical processing of patients brain tissue

Paraffin-embedded brain tissues were sectioned transversely at a thickness of 4 µm. The sections were subsequently deparaffinized and subjected to antigen retrieval using a citrate buffer. Following this, sections underwent three washes with PBS 1 × for 5 min each. Endogenous peroxidases were blocked for 2 h using a solution containing 10% methanol, 2% H₂O₂, and PBS 1x, adjusted with distilled water. After three additional washes with PTB buffer (PBS 1x, 0.02% Triton X-100, and Bovine Serum Albumin [BSA]), sections were washed three times with PBS 1 × for 5 min each. Autofluorescence was quenched using TrueBlack^®^ Lipofuscin Autofluorescence Quencher (Biotium), followed by three more washes with PBS 1 × . Non-specific epitopes were blocked for 90 min using 5% Normal Goat Serum and 0.01% BSA in PBS 1x.

Tissue sections were incubated for 5 days at 4 °C with primary antibodies against PLIN2 (1:200, PA5-29099, Thermo Fisher Scientific), TDP-43 (1:250, 10782-2-AP, Proteintech) and IBA1 (1:400, AB300157 Abcam) diluted in PBS 1 × with 0.01% BSA. After three 5-min washes with PBS, sections were incubated overnight with Alexa Fluor 594- and 488-conjugated secondary antibodies, respectively (1:400 dilution, Thermo Fisher Scientific), and the nuclei were stained with DRAQ5^™^ (1:1500, 65-0880-92, Invitrogen). Z-section images were taken using confocal microscopy Olympus Fluoview FV1000 v.3.1.1.9. Images were loaded into ImageJ v.1.53q for analysis and intensity quantification. For the total intensity protocol, we converted the image to binary; then we quantified the number of particles (analyze particles) by selecting the IntDen function of ImageJ/Fiji cell by cell that were IBAI1 positive. The total number of particles was then multiplied by the IntDen value to obtain the total intensity value. This protocol was used for the analysis of PLIN2.

##### Mouse brain immunostainings

Female mice, both *Tardbp*^+*/*+^ and *Tardbp*^*M323K/M323K*^ (n = 4 or 5 per group), were terminally anesthetized with 0.4 mg/kg of fentanest and 40 mg/kg of thiopental and cardiac perfused with the fixative 4% PFA without methanol in PBS 1x, pH 7.4. The dissected brains were post-fixed in the same fixative overnight at 4 °C and kept on PBS 1 × with sodium azide 0.1% until use. Whole brains were cryopreserved by immersion in 30% sucrose. Brains were dried and embedded in OCT using isopentane and dry ice, and frozen at − 20 °C until they were sectioned in a cryostat (Leica). Coronal cryosections at 30 μm were collected free-floating through the brain frontal cortex or the cerebellum. Brain sections were selected using anatomical references from the Paxinos and Franklin mouse brain atlas (interaural 4.78 mm, bregma 0.98 mm). For the analyses, we focused on the M1 motor cortex, particularly the area adjacent to the corpus callosum, as a consistent anatomical landmark. Free-floating sections were then blocked with PTB (PBS 1x, Triton 0.02% and BSA) and then incubated with the primary antibodies overnight at 4 °C, PLIN2 (1:300, PA5-29,099, Thermo Fisher Scientific), PLP1 (1:400, AB254363, Abcam), TDP-43 (1:250, 67345-1-Ig, Proteintech and 1:250, MA532627, Thermo Fisher Scientific), and IBAI1 (1:250, ab5076, Abcam) in PBS with 0.02% Triton X-100 (PBS-T). After three washes of PBS-T, the secondary antibodies Alexa-Fluor conjugated -488 and -594 (1:400) were incubated for 4 h, and the Hoechst 33,342 (1:1000, H21492, Thermo Fisher Scientific) stain was used to detect nuclei. Z-section images were taken using a confocal microscopy Olympus Fluoview FV1000 v.3.1.1.9, quantifying at least three to five photos that contain at least 300 cells per animal. Confocal Z-stack images were uploaded into ImageJ v.1.53q for the analysis: intensity quantification, co-localization, and cell number counts. For the total intensity protocol, we converted the image into binary, then we quantified the number of particles (analyze particles) by selecting the IntDen function of ImageJ/Fiji. Subsequently, the total number of particles was multiplied by the IntDen value, obtaining the total intensity value. This protocol was used to analyze PLIN2, PLP1, and TDP-43. The ‘Colocalization Threshold’ macro in the Fiji software was used to calculate the percentage of co-localization. This protocol was used to analyze TDP-43 co-localization versus PLIN2 and versus IBA1. Co-localization analysis was performed using the Colocalization Threshold plugin in the Fiji (ImageJ) software package. Prior to analysis, background subtraction was performed on all multi-channel RGB.oib images (rolling ball radius: 50 pixels) to enhance signal specificity. The z-stack of confocal images had a thickness of 8 microns, with images taken at 0.75-micron intervals. The Costes’ threshold algorithm was then applied to determine the optimal intensity threshold for each fluorophore channel objectively. This iterative method, which was performed 100 times with a significance level of 0.05, ensures that co-localization is assessed only from the genuine signal and not from background or random overlap.

The primary quantitative metrics reported were the Manders’ overlap coefficients (TM1 and TM2). TM1 quantifies the fraction of the total intensity of Channel 1 pixels that colocalize with positive pixels in Channel 2. Conversely, TM2 represents the fraction of Channel 2’s total intensity that overlaps with Channel 1. These threshold coefficients provide robust, intensity-independent measures of the extent of true fluorophore co-localization within specific regions of interest.

##### Fibroblasts immunostainings

Following the 24 h treatment, the fibroblasts were washed with PBS and fixed with 4% (w/v) paraformaldehyde without methanol in PBS for 15 min at room temperature. For the LD staining, fibroblasts were first incubated with the primary antibody of SREBP2 (1:200, PA1-338, Thermo Fisher Scientific) overnight at 4 °C. After three washes with PBS, the cells were incubated for 1 h at room temperature with the secondary antibody conjugated with fluorophores (1:1500 dilution, Alexa fluor 594 and 647, Thermo Fisher Scientific). Nuclei were stained with HOESCHST 33342 (1:3000 dilution, 11,504,886/H21492, Fisher scientific) and neutral lipids from LDs were stained with BODIPY^493/503^ (1:1000 dilution) for an extra 30 min. A final three washes with PBS were done to remove the excess, and the coverslips were mounted on a slide with FluorSave Reagent (Merck). Images were taken using a confocal microscope Olympus Fluoview FV1000 with a version system 3.1.1.9, quantifying at least five photos that contained at least 50 cells per animal with ImageJ v.1.53q.

##### Western blot

Male mice, both *Tardbp*^+*/*+^ and *Tardbp*^*M323K/M323K*^, at 3 and 12 months of age (*n* = 3 or *n* = 4 per group, respectively), were sacrificed, and fresh brains were taken on ice. To dissect the frontal cortex from fresh tissue, the olfactory bulbs were first removed. A coronal cut was made at bregma 0, selecting all the anterior cortex carefully excluding the striatum. The two hemispheres were then separated. Fresh dissected tissues were snap frozen and stored at − 80 °C until required. Frontal cortex from one hemisphere was homogenized using mechanical disaggregation with beads (Precells) in RIPA buffer (Thermo Fisher Scientific) at 4 °C with protease and phosphatase inhibitors cocktail (Roche). After 20 min of centrifugation at 13000 g at 4 °C, the supernatant was collected, and the total amount of protein was quantified using the DC Assay method (Bio-Rad). 30 μg of total protein was loaded for each of the samples in a 10% bis-acrylamide precast gel (GenScript, US) under reducing conditions, run and transferred into a PVDF low fluorescence membrane (GE healthcare). The membranes were blocked for 1 h and incubated with the primary antibodies overnight: SREBP2 (1:200, PA1-338, Thermo Fisher Scientific), MOG (1:1000, AB243034, Abcam), and PLP1 (1:2000, AB254363, Abcam). Secondary antibodies labeled for infrared detection were scanned in the infrared scanner Clx (LiCor). The total protein detection kit was used following manufacturer’s instructions (LiCor) for the loading correction. The intensity of the bands was quantified using the image software Image Studio v.5.2 (LiCor).

#### Fibroblasts flow cytometry for LD analysis

Primary fibroblast from wild-type and homozygous mice were cultured in six-well plates for at least 24 h and then we performed a treatment with oleic acid 250 µM for 24 h. Following treatment, cells were stained with BODIPY^493/503^ (1:1000 dilution, Thermo Fisher Scientific) and incubated for 1 h and 30 min. After incubation, cells were washed with PBS and detached using trypsin for 3–5 min at 37 °C. The reaction was then halted by adding culture medium, and cells were centrifuged at 1000 rpm for 5 min. The supernatant was discarded, and the pellet was resuspended in PBS. This washing step was repeated once more by centrifugation at 1250 g for 5 min, followed by resuspension in 300 µL of PBS. Flow cytometry analysis was performed using a FC500 flow cytometer (Beckman Coulter) analyzing at least 10,000 events, and results were processed using FlowJo Software (BD Life Sciences) v.10.8.1.

#### Lipidomic analysis

As previously described in Sect.  "Western blot", we used the dissected frontal cortex from one hemisphere from *Tardbp*^+*/*+^ and *Tardbp*^*M323K/M323K*^ male mice (*n* = 6 per group) at 9 months of age for the lipidome analysis. Briefly, brain tissues were treated using the modified Bligh and Dyer method for lipid extraction. The homogenates were vortexed for 15 s, then incubated at 4 °C on a mixer (300 rpm) for 1 h. After agitation, 300 μl of ice-chilled chloroform and 250 μl of ice-chilled MQ water were added sequentially, followed by vortexing for 15 s, and centrifugation at 9,000 rpm for 2 min to separate the phases. Bottom organic phases were collected, and the aqueous phases were re-extracted with 500 μl of chilled chloroform. Collected organic phases were dried in a vacuum concentrator and stored lyophilized at − 80 °C.

Samples were then solubilized in 100 μl chloroform/methanol (1:1; v/v) and analyzed in reverse phase using an Acquity UPLC HSS T3 1.8 μm column with the following conditions: mobile phase A (water:acetronitrile, 40:60, with 10 μM ammonium acetate and 0.1% acetic acid), B (water:acetonitrile:isopropanol:acetic acid, 5:10:85:0.1, with 10 μM ammonium acetate and 0.1% acetic acid); flow rate of 300 μl/min; injection volume of 5 μl; column temperature at 55 °C; 20% B for 1.5 min; linear change to 100% B over next 16.5 min; and maintained at 100% B for 3 min (77). Then the gradient was reverted back to initial state 20% B for 1 min, and then held for next 1 min at 20% B. QC samples were injected prior to, and after every five samples, to monitor the stability of the instrument. Samples were run under untargeted positive and negative electrospray ionization (ESI) modes in a data‐independent manner (MSE mode). The following ESI conditions were used: for positive, capillary at 2 kV, sampling cone at 35 V, source temperature at 100 °C, desolvation gas at 500 l/h, and nebulizer at 6.5 bar; and for negative, capillary at 2.2 kV, sampling cone at 40 V, source temperature at 80 °C, desolvation gas at 500 l/h, and nebulizer at 6.5 bar. For lock mass correction, leucine enkephalin was used at 1 ng/ml in acetonitrile/water (1:1, v/v) with 0.1% formic acid and at a flow rate of 10 μl/min. The low collision energy was set to 4 eV and high collision energy was set between 25 and 40 eV for both positive and negative modes. Pooled samples were run at first and every after six samples as QC. Raw data were converted into ABF format using Reifycs Analysis Base File Converter, and then used in MS‐Dial (v. 4.9) for peak picking and retention time alignment using default settings. Lipid species were manually verified and named using Lipid Maps abbreviations. The intensities were initially normalized with total ionic current. The corrected readings of identified species were exported into R (v. 4.4.2) to do the batch correction using pooled samples as QC, and then calculated the concentrations using the known concentration of spiked internal standards (Avanti Polar Lipids, Alabaster, AL). Lipid levels for each sample were calculated by summing up the total number of moles of all lipid species measured by all three LC–MS methodologies and then normalizing the total to mol %.

For the lipidomic statistical analysis (single factor), we used MetaboAnalyst 6.0 (https://www.metaboanalyst.ca/ accessed on 3 February 2025). Normalization by sum and Pareto scaling were used. Traditional univariate methods (volcano plot), multivariate statistics (partial least squares discriminant analysis (PLS-DA)), and clustering (heat map) were provided. LION was used to identify altered lipid functions and organelle associations (https://www.lipidontology.com/ accessed 5 February 2025).

#### RNA study

##### RNA extraction from mouse frontal cortex

The RNA was extracted from the snap frozen frontal cortex from one hemisphere, dissected as described in Sect. "Western blot", of *Tardbp*^+*/*+^ and *Tardbp*^*M323K/M323K*^ male mice at 9 months of age (*n* = 4 per group). The tissue was homogenized with mechanical disaggregation in tubes with ceramic beads (Prescells) in 400 µl of Trizol. The RNA was extracted in columns using the RNeasy Lipid Tissue Mini Kit (Qiagen) and removed potential genomic DNA contamination with DNAsa treatment in the columns (Qiagen), following the manufacturer’s instructions. The quantity and quality of the RNA were measured before use.

##### Gene expression analysis

cDNA synthesis was performed using the High-Capacity cDNA Reverse Transcriptase Kit (Thermo Fisher Scientific) from 1 μg of total RNA. cDNA for qPCR reactions was used at a final concentration of 25 ng per well using Power SYBR^™^ Green PCR Master Mix (Thermo Fisher Scientific) and the appropriate pair of primers in a final well volume of 10 μl per well, in duplicates. The housekeeping gene used was the hypoxanthine phosphoribosyltransferase *(Hprt*) gene.

A list and sequences of all primers used can be found in Supplementary Table 1.

##### RNA sequencing

We use the RNA that was previously extracted as described in Sect. "RNA extraction from mouse frontal cortex" of *Tardbp*^+*/*+^ and *Tardbp*^*M323K/M323K*^ male mice at 9 months of age (*n* = 4 per group). Quality and quantity were assessed using the RNA Nano 6000 Assay Kit of the Bioanalyzer 2100 system (Agilent Technologies). Sequencing libraries were generated using an NEBNext^®^ Ultra ™ RNA Library Prep Kit for Illumina^®^ (NEB) following the manufacturer’s recommendations, and index codes were added to attribute sequences to each sample. The clustering of the index-coded samples was performed on a cBot Cluster Generation System using PE Cluster Kit cBot-HS (Illumina) according to the manufacturer’s instructions. After cluster generation, the library preparations were sequenced on an Illumina platform, and 150 bp paired-end reads were generated. Library preparation and RNA-seq were carried out by Novogene. Quality control of FastaQ files was performed using FastQC (https://www.bioinformatics.babraham.ac.uk/projects). Low-quality reads (Phred quality score < 30) and reads too short (length < 15 pb) were removed using Fastp [6]. The alignment to the genome (mm39 mouse reference genome from NCBI refseq) was achieved using HISAT2 [23].

##### DEG expression analysis of the transcriptome

The expression quantification of genes was carried out using FeatureCounts [25]. Only uniquely mapped reads were used for the analysis of differential gene expression quantification with DESeq2 [27]. Raw *p *values were adjusted by the Benjamini–Hochberg false-discovery rate (FDR) method and the adjusted *p *values or *p *value less than 0.05 were considered statistically significant. Functional enrichment was done by the Over-Representation Analysis (ORA) [22] method, which performs a statistical evaluation of the fraction of genes in a particular pathway found among the set of genes. A protein–protein interaction network was constructed using STRING [42] from the different comparison we did and the DEGs found. The following parameters were selected: network type: full STRING network; required score: medium confidence (0.4); FDR stringency: medium (5 percent).

##### Splicing analysis

After alignment of RNA-seq reads by HISAT2, alternative splicing events were identified and quantified by rMATS-turbo 4.3.0 using gene transcript annotations from NCBI refseq mus musculus version mm39 reference genome. rMATS was downloaded from the open-source platform SourceForge (https://rnaseq-mats.sourceforge.io/, accessed on 10 December 2023) [40] and rmats2sashimiplot was used to convert the rMATS output into Sashimi plot, including novel SS events. The cut-off criteria for statistical significance were adjusted *p* value < 0.05 and |IncLevelDifference|> 5%. A Venn diagram was used in splicing and transcriptomic analysis to determine which genes or genes with splicing events were common between two or more lists of genes [51].

##### Analysis of RNA sequencing database from FTLD patients and controls

The data used in this study of the frontal cortex transcriptomics from FTLD-TDP patients were obtained from the supplementary file available in this article published in Brain [35]. The data were downloaded, and using RStudio v4.3.3., we performed the analysis of differential gene expression quantification with DESeq2 of the frontal cortex samples comparing FTLD type A, B or C with the control patients. The different FTLD patients were categorized as follows: Type A is often associated with GRN mutations, presenting with bvFTD and sometimes non-fluent variant PPA. Type B is linked to patients with *C9ORF72* expansions, characterized by bvFTD with ALS. Type C, associated with TDP-43 pathology, is common in patients without MAPT mutations and is strongly associated with semantic variant PPA. Finally, we selected the fold-change value of the desired genes and were plotted in GraphPad Table 2.

**Table 2:** Patients demographics data for the transcriptomic analysis

	**Age**	**N Total**	**Sample type**
Control	28–100	24	Frontal cortex
FTLD-TDP-A	67–94	24	Frontal cortex
FTLD-TDP-B	49–97	20	Frontal cortex
FTLD-TDP-C	61–83	22	Frontal cortex

### Statistical analyses

Statistical analysis was conducted using GraphPad Prism. Two groups were compared with a single time point using Student’s *t *test. Body weights between genotypes, across multiple time points, were compared using two-way ANOVA repeated measures/mixed models and Bonferroni correction of multiple testing. Two groups were compared across multiple time points using two-way ANOVA with Sídak’s multiple comparisons post hoc test. Three or more groups were compared at a single time point using one-way ANOVA with Dunnett’s post hoc test. Statistical analysis of qRT-PCR data was performed on ΔCT values. Two- way ANCOVA (SPSS) was used to correct the energy expenditure data for the lean mass interaction. Statistical significance was defined as *p* ≤ 0.05 for analysis of phenotyping and molecular biology data. Please see figure legends for sample size—*n* numbers; n numbers refer to biological samples (i.e., number of animals used in animal experiments). Statistical detail for each experiment can be found in figure legends. Where indicated the following as: * = *p* ≤ 0.05, ** = *p* ≤ 0.01, *** = *p* ≤ 0.001, **** = *p* ≤ 0.0001. All graphs were generated using GraphPad Prism 8. BioRender.com was used to create original diagrams/figures.

## Results

### ***Tardbp***^***M323K/M323K***^*** mice display alterations in myelin proteins composition in the frontal cortex***

**Fig. 1:**
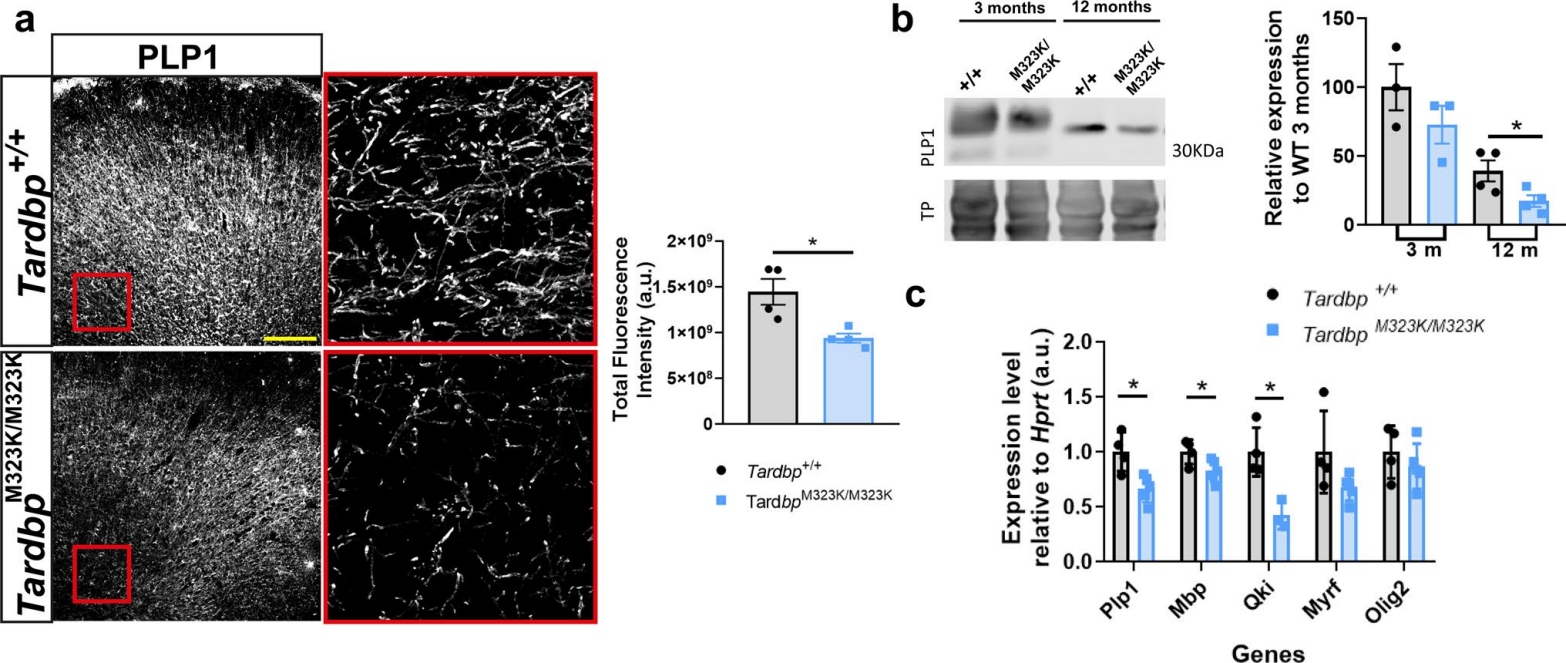
Reduction of myelin proteins and oligodendrocyte-related gene expression in *Tardbp*^*M323K/M323K*^ mice. **a** Representative images of PLP1 immunostaining in the frontal cortex of *Tardbp*^+*/*+^ and *Tardbp*^*M323K/M323K*^ mice, with quantification of total fluorescence intensity (right). Scale bar: 100 µm. Experimental n per group: 4 *Tardbp*^+*/*+^ and 4 *Tardbp*^*M323K/M323K*^. **b** Western blot analysis of PLP1 protein levels in frontal cortex samples with quantification relative to wild-type at 3 months. Experimental n per group: 3 *Tardbp*^+*/*+^ and 3 *Tardbp*^*M323K/M323K*^ of 3 months and 4 *Tardbp*^+*/*+^ and 4 *Tardbp*^*M323K/M323K*^ of 12 months. **c** Relative expression of myelin- and oligodendrocyte-related genes (*Plp1, Mbp, Qki, Myrf, Olig2*) normalized to *Hprt* in frontal cortex samples from *Tardbp*^+*/*+^ and *Tardbp*^*M323K/M323K*^ mice. Experimental n per group: 4 *Tardbp*^+*/*+^ and 5 *Tardbp*.^*M323K/M323K*^ of 3 months. All are females. Data are presented as mean ± SEM. *p < 0.05, **p < 0.01

We have previously shown that the M323K mutation in TDP-43 affected the myelin formation and its progressive stability in the homozygous mice [17] macroscopically. Here, we further evaluated the impact of the M323K mutation on myelination at the molecular level, conducting an analysis of the expression levels of key myelin-related proteins and genes in the frontal cortex of wild-type and homozygous mutant mice at different ages. The myelin-associated protein PLP1 (Proteiolipid protein 1) immunofluorescence staining revealed a significant reduction in the frontal cortex of the *Tardbp*^*M323K/M323K*^ mice compared to their *Tardbp*^+*/*+^ littermates (Fig. 1a). Western blot analysis confirmed the reduction in PLP1 protein levels in the *Tardbp*^*M323K/M323K*^ frontal cortex, which significantly declined in the *Tardbp*^*M323K/M323K*^ mice at 12 months compared to the wild-type controls (Fig. 1b), as well as a trend decrease in the expression levels of MOG, another myelin-associated protein (Supplementary Fig. 1a). The progressive reduction of these myelin-associated proteins supports the potential effect of TDP-43 mutation in impairing myelin maintenance. Next, we looked at the expression level of key oligodendrocytes and myelin-related genes in the frontal cortex of mutant mice. The expression levels of the genes *Plp1*, *Mbp*, *Qki*, and *Myrf* were significantly downregulated in *Tardbp*^*M323K/M323K*^ mice compared to their *Tardbp*^+*/*+^ littermates, whereas *Olig2* expression remained unchanged (Fig. 1c and Supplementary Fig. 1b). This could indicate that TDP-43-M323K mutation could be interfering downstream to the oligodendrocyte precursor cells (OPCs) production, affecting the transcriptional regulation of genes essential for myelin maintenance.

### ***Disrupted lipids landscape in the frontal cortex of the Tardbp***^***M323K/M323K***^*** mice***

**Fig. 2:**
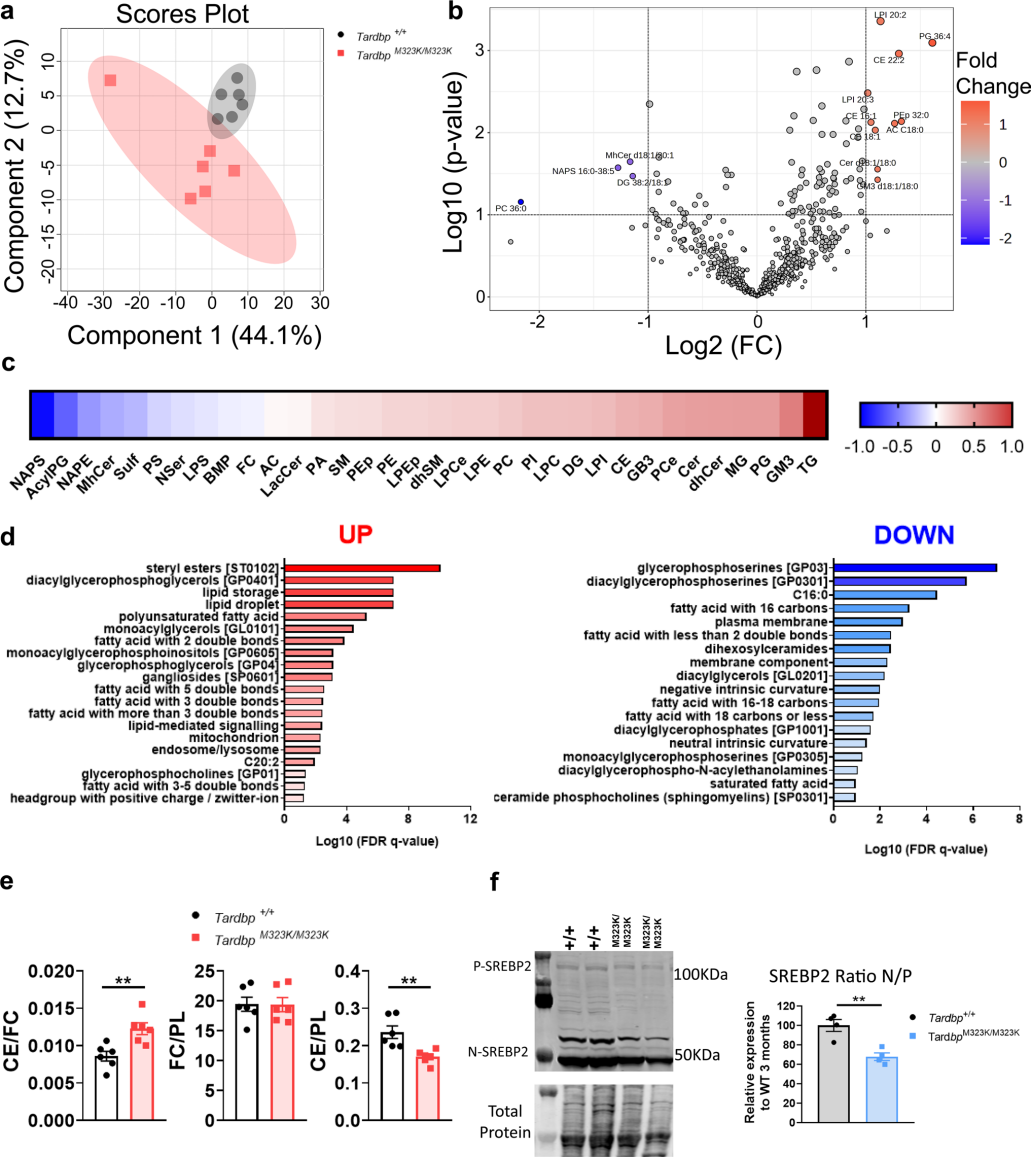
*Tardbp* altered lipid metabolism in the frontal cortex. **a** Partial least squares discriminant analysis (PLS-DA) of samples lipidome. **b** Volcano plot analysis on lipids. Red dots indicate significantly upregulated genes, blue dots indicate significantly downregulated genes, and gray dots indicate genes with no significant change. **c** Enrichment analysis of the frontal cortex in the “ranking mode” The gray vertical lines indicate the cut-off value of significant enrichments (*q* < 0.05). Bar colors are scaled with the enrichment (− log q-values). FDR: false-discovery rate. **d** Lipid Ontology (LION) enrichment analysis with upregulation in lipid droplets and cholesterol esters, together a downregulation in membrane components **e** Ratio of cholesteryl ester:free cholesterol:total glycerophospholipids and cholesteryl ester:total glycerophospholipids. Male mice at 9 months of age, n = 6 *Tardbp*^+*/*+^ n = 6 *Tardbp*^*M323K/M323K*^ in lipidomics data.** f** Western blot analysis of SREBP2 protein levels in frontal cortex samples at 12 months of age, with quantification relative to wild-type (WT). Female mice, *n* = 4 *Tardbp*^+*/*+^ *n* = 4 *Tardbp*^*M323K/M323K*^*.* Data are presented as mean ± SEM. **p < 0.01

We hypothesized that the myelin alterations observed might, at least partly, be driven by disruptions in lipid metabolism, and that TDP-43 dysfunction could be impairing the regulation of brain lipid homeostasis. To this end, we performed a targeted lipidomic analysis of the frontal cortex of *Tardbp*^*M323K/M323K*^ and wild-type littermate mice (*n* = 6) at 9 months of age. Partial least squares discriminant analysis (PLS-DA) (Fig. 2a) showed that 44% of the variability between samples was due to the mutation, and the metabolites that contributed most to this component (VIP > 1) (Supplementary Fig. 2a) were cholesterol, cholesterol ester (CE) species, and some dihydrosphingomyelin species. We found a total of 14 metabolites differentially expressed in the frontal cortex of mutant mice compared to wild-type controls, with log2 fold-change (log2 (FC)) values between − 1 and + 1 and -log10 (*p* value) > 1. A total of ten metabolites (LPI 20:2, LPI 20:3, PG 36:4, CE 22:2, CE 16:1, CE 18:1, PEp 32:0, AC C18:0, Cer d18:1/18:0 and GM3 d18:1/18:0) were upregulated and four downregulated (MhCer d18:1/20:1, NAPS 16:0–38:5, DG 38:2/18:1 and PC 36:0) (Fig. 2b). Among the top significant hits, we identified lipid species of three main families, i.e., the neutral lipids (CE, DG and AC), sphingolipids (Cer, Mhcer and GM3), and phospholipids (Pep, PC, PG, LPI and NAPS), highlighted in the volcano plot (Fig. 2b).

Looking closer at the particular perturbation of the major lipid classes by fold change in the mutant brains compared with the wild type, the redistribution of lipids suggested changes possibly related to alterations in membrane composition (sphingolipids) and lipid droplet formation (TGs and CEs) (Fig. 2c). These results were more clearly illustrated when we examined the 25 most altered lipids, based on fold change, in a heatmap (Supplementary Fig. 2b), showing that triglycerides and cholesterol esters were the most increased in the mutant mice compared to their wild-type littermates. To investigate this further, we performed a Lipid Ontology (LION) enrichment analysis, which concordantly revealed an increase in lipid droplets, likely driven by elevated TG and CE, and a reduction in membrane components, associated with a reduction in free cholesterol (FC) and changes in sphingolipids (Fig. 2d).

Cholesterol esterification is a process that increases the hydrophobicity of this lipid, thereby facilitating its storage or transport. This mechanism prevents cellular toxicity caused by high levels of free cholesterol in the membrane. From the lipidomic analysis, we determined the rate of cholesterol esterification, the fractional cholesterol esterification rate (FCE, as the ratio between CE and FC). An increase in this ratio was observed in the mutant mice compared to the wild type (Fig. 2e). There was also a significant decrease in CE compared to membrane phospholipids (the ratio between CE and glycerophospholipids (PL)) (Fig. 2e), which may be indicative that there was a reduction in cholesterol levels in cellular membranes in the *Tardbp*^*M323K/M323K*^ mice.

We looked deeper into the regulation of cholesterol biosynthesis in the frontal cortex of these mice in particular through SREBP2, which is retained in the endoplasmic reticulum (ER) in an inactive form bound to SCAP and INSIG proteins. When cholesterol levels drop, SCAP escorts SREBP2 to the Golgi, where it undergoes proteolytic cleavage, releasing its active fragment. This active form translocates to the nucleus, where it binds to sterol regulatory elements (SREs) in target gene promoters, inducing the expression of genes involved in cholesterol biosynthesis (HMG-CoA reductase (HMGCR), HMG-CoA synthase (HMGCS), and mevalonate kinase (MVK) and DHCR24). SREBP2 activation was measured by the ratio of the nuclear fragment to precursor SREBP2 protein. The ratio of active SREBP2 seems to decrease at 12 months of age in the brain of the *Tardbp*^*M323K/M323K*^ mice compared to their *Tardbp*^+*/*+^ littermates, which could explain the decreased amounts of free cholesterol observed in the mutants brains.

Altogether, the TDP-43 M323K mutation caused a clear dysregulation of lipids, in particular an imbalance of cholesterol-related species in the frontal cortex of mice, that was an increase in the storage of CE and TG in the form of lipid droplets, while reducing the synthesis of FC and the dysregulation of other lipids involving membrane components (sphingolipids and phospholipids).

### Altered TDP-43 induced accumulation of lipid droplets (LD) in cells and in the frontal cortex of mice and FTLD-TDP patients

The lipidomic analysis of the frontal cortex from the mutant TDP-43 mice revealed an increase in the neutral lipids CE and TGs, which are normally accumulated in the cells in the form of lipid droplets (LD). Consequently, we aimed to investigate whether alterations in the TDP-43 protein, under various conditions and across different tissues in both mice and humans, supported these findings.

**Fig. 3:**
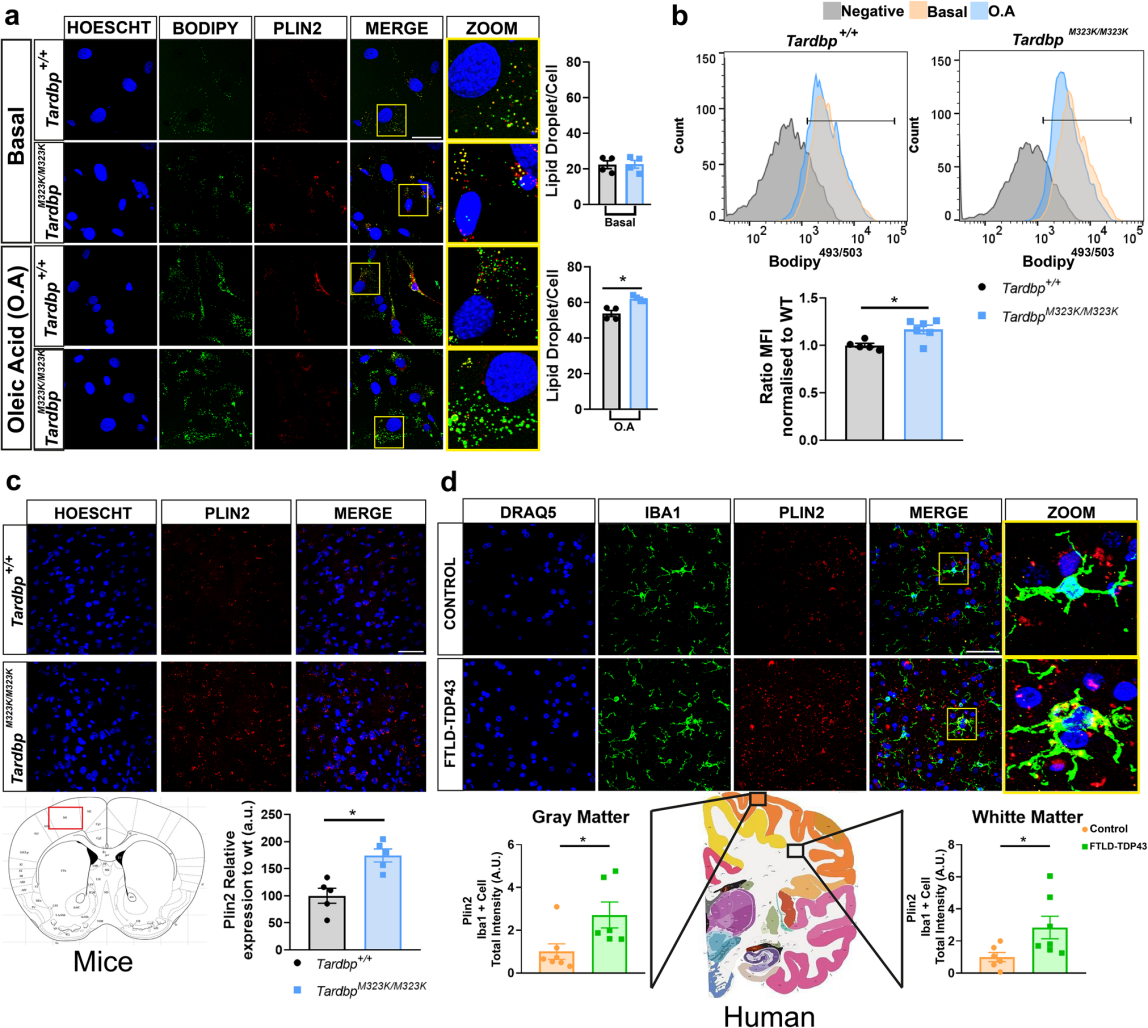
Increased lipid droplet accumulation and PLIN2 expression in *Tardbp*^*M323K/M323K*^ mice and FTLD-TDP43 patients.** a** Representative images of lipid droplets in fibroblast cultures from *Tardbp*^+*/*+^ and *Tardbp*^*M323K/M323K*^ mice under basal conditions and after oleic acid (O.A.) treatment. Cells were stained with Hoechst (nuclei, blue), BODIPY (lipid droplets, green), and PLIN2 (red). Merged images highlight co-localization. Quantification of lipid droplet number per cell (bottom). Scale bar = 20 µm. Experimental n per group: 4 *Tardbp*^+*/*+^ and 4 *Tardbp*^*M323K/M323K*^. **b** Flow cytometry analysis of lipid content in fibroblasts from *Tardbp*^+*/*+^ and *Tardbp*^*M323K/M323K*^ mice, with quantification of the ratio of mean fluorescence intensity (MFI) normalized to wild-type (WT) (bottom). Experimental n per group: 5 *Tardbp*^+*/*+^ and 6 *Tardbp*^*M323K/M323K*^. **c** Representative images of Plin2 expression in frontal cortex sections from *Tardbp*^+*/*+^ and *Tardbp*^*M323K/M323K*^ mice, with quantification (bottom). Experimental n per group: 5 *Tardbp*^+*/*+^ females and 5 *Tardbp*^*M323K/M323K*^ females. Scale bar = 20 µm. **d** Representative images of PLIN2 expression in brain sections from control and FTLD-TDP43 patients. Sections were stained with DRAQ5 (nuclei, blue), IBA1 (microglia, green), and PLIN2 (red). Quantification of PLIN2 total intensity in gray and white matter (bottom). Scale bar = 20 µm. Experimental n per group: 6 controls and 6 FTLD-TDP43 patients. Data are presented as mean ± SEM. *p < 0.05

Since the analyses of lipid droplets are very challenging in neuronal tissue, as well as being a dynamic process, we first investigated the impact of the TDP-43 M323K mutation directly in the process of lipid droplets intracellular accumulation in a cell-autonomous system using primary cells derived from these mice. Thus, we generated primary fibroblasts from the ears of 3-month-old *Tardbp*^+*/*+^ and *Tardbp*^*M323K/M323K*^ mice and challenged them with oleic acid to evaluate if the mutation could directly intervene in the cell lipid storage capacity. We stained the lipid droplets with the marker BODIPY and quantified the positive staining by confocal microscopy (Fig. 3a). There were no differences between the two groups at baseline; however, after a 24 h challenge with oleic acid, the number of lipid droplets per cell, stained with BODIPY, was higher in cells from the *Tardbp*^*M323K/M323K*^ mice compared to those from the *Tardbp*^+*/*+^ mice. We corroborated these results quantifying the BODIPY fluorescence in the fibroblasts by flow cytometry and confirmed that the ratio of mean fluorescence intensity (MFI) obtained between oil-treated and BSA-untreated cells was higher in *Tardbp*^*M323K/M323K*^ cells (Fig. 3b).

Next, we sought to confirm whether this alteration in LD levels could be observed in the brain of the mice. Direct staining of lipid droplets using BODIPY in the brain, which is extremely rich in lipids, is not a reliable and sensitive technique. Thus, we performed immunohistochemical staining of one of the proteins that coats LD in the nervous system, the protein PLIN2 (perilipin 2), in the frontal cortex of 3-month-old mice. PLIN2 assists with the storage of neutral lipids in the LDs and is highly expressed in neurons and greatly affected by age [10] Even at early ages, the mutant TDP-43 protein induced a higher amount of PLIN2 protein in the frontal cortex (Fig. 3c), which denoted increased LD accumulation in those mutant brains, as expected from the lipidomic analysis (Fig. 2d). In the mutant brains, the amount of PLIN2 immunoreactivity was higher in the IBA1 cells (Supplementary Fig. 3 and supplementary video).

Furthermore, we evaluated whether the increased LD accumulation was a specific effect of the particular M323K mutation in TDP-43 or whether it is a more general effect of TDP-43 alterations, as in the case of the FTLD-TDP cases without a *TARDBP* mutation. Hence, we evaluated the staining of PLIN2 in postmortem frontal cortex white and gray matter tissue from FTLD-TDP patients and non-neurological diseased age–sex matching controls (*n* = 7 per group) (Table 1). The PLIN2 staining in the postmortem human tissue was not only intracellular, so we performed a co-staining with the cellular microglia marker IBA1 with PLIN2, both in white and gray matter. The quantification of the microglial-intracellular PLIN2 was higher in both the gray and white matter of the frontal cortex of FTLD-TDP patients compared to the controls (Fig. 3d). The TDP-43 pathological evaluation was confirmed using anti phospho-TDP43 in the serial sections (data not shown). The results were similar across the different subtypes of TDP-43 proteinopathies.

**Fig. 4:**
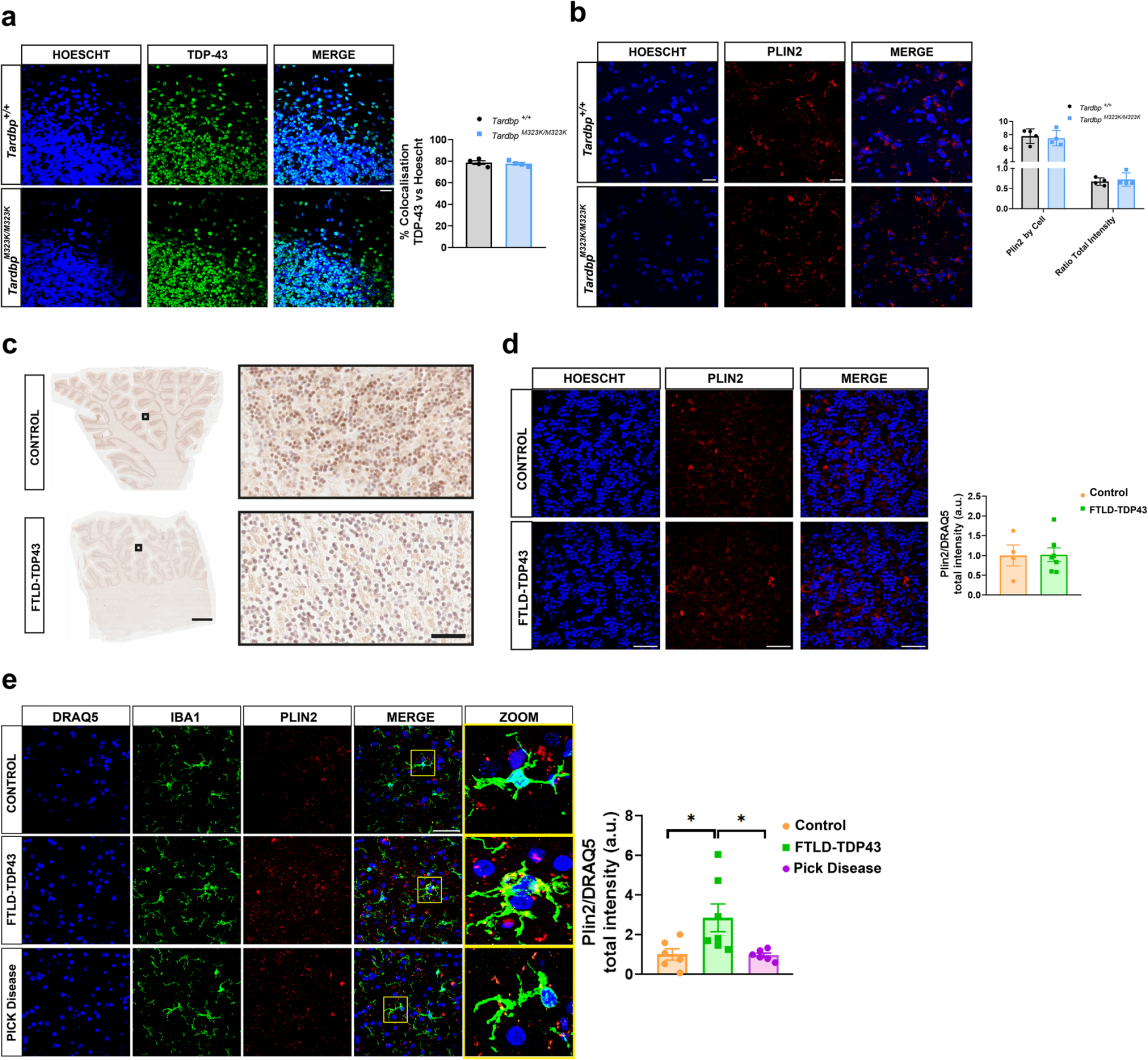
PLIN2 immunoreactivity in the cerebellum of *Tardbp*^*M323K/M323K*^ mice as well as in the cerebellum and frontal cortex of FTLD-TDP43 and Pick’s patients.** a** Immunofluorescence for TDP-43 in the cerebellum from *Tardbp*^+*/*+^ and *Tardbp*^*M323K/M323K*^ mice showed no significant change in nuclear localization of TDP-43 (*n* = 4 per group). **b** Immunofluorescence for PLIN2 in the cerebellum of these mice. **c** Representative immunohistochemistry for TDP-43 in the cerebellum of control and FTLD-TDP patients. **d** Immunofluorescence for PLIN2 in human control and FTLD-TDP cerebellums. Quantification reveals no differences in PLIN2 signal in FTLD-TDP samples with the control group (*n* = 4–7 per group). **e** Triple immunofluorescence staining for IBA1 (microglia), PLIN2, and DRAQ5 in human control, FTLD-TDP, and Pick’s disease brain samples. High-magnification (ZOOM) images show PLIN2 signals localized within microglia. Quantification of PLIN2 intensity relative to nuclear area (PLIN2/DRAQ5) reveals a significant increase in FTLD-TDP compared to control and Pick’s disease cases (*n* = 4–7 per group). Data are presented as mean ± SEM. *p* < 0.05, one-way ANOVA or unpaired *t *test as appropriate. Scale bars: 100 μm (A, overview), 20 μm (B–E, and A, insets)

To evaluate whether the increase in immunoreactivity of PLIN2 was associated to TDP-43 pathology, we further analyzed the levels of PLIN2 in the cerebellum of the same mutant mice and FTLD-TDP patients, where TDP-43 pathology has not been reported. First, we analyzed the subcellular localization of TDP-43 in the cerebellum of TDP-43 homozygous mice. Interestingly, in contrast to the mislocalization of TDP-43 to the cytoplasm observed in the frontal cortex and hippocampus, no cytoplasmic TDP-43 was detected in the cerebellum (Fig. 4a). Consistently, PLIN2 immunoreactivity in the cerebellum of these mice was comparable to that observed in control animals (Fig. 4b). To validate these findings, we examined cerebellar tissue from the same FTLD-TDP patients analyzed in Fig. 3d. As expected, no pathological TDP-43 staining was observed in the cerebellum (Fig. 4c), and PLIN2 immunoreactivity remained at levels similar to those in the control group (Fig. 4d). Lastly, we examined whether PLIN2 immunoreactivity was also present in non-TDP-43-related FTLD cases, such as the frontal cortex of Pick’s disease (FTLD-tau). While lipid droplet formation and increased PLIN2 expression are not exclusive to TDP-43 pathology, we observed that PLIN2 levels were significantly higher in the frontal cortex of FTLD-TDP patients compared to those with FTLD-tau (Fig. 4e).

In summary, these results demonstrated that alterations in TDP-43 resulted in increased LD formation in the cortex of mutant mice, which was consistent with the lipidomic data obtained. This effect was also observed in the postmortem frontal cortex of FTLD-TDP patients without a *TARDBP* mutation. Moreover, in the cerebellum of mutant mice and FTLD-TDP patients where there was no TDP-43 pathology, as in the frontal cortex of other non-TDP-43 FTLD patients, the levels of immunoreactivity of PLIN2 were similar to those of the controls, supporting the influence of TDP-43 alteration in LD and the associated PLIN2 LD marker increase. Importantly, our findings showed that the TDP-43 alteration influences LD formation not only as a consequence of the neurodegenerative disease in the brain, confirmed through a lipid storage challenge in a cell-autonomous system, although further in vitro molecular experiments are needed to confirm these results.

### Disrupted cholesterol metabolism in patients and mice with TDP-43 proteinopathy

**Fig. 5:**
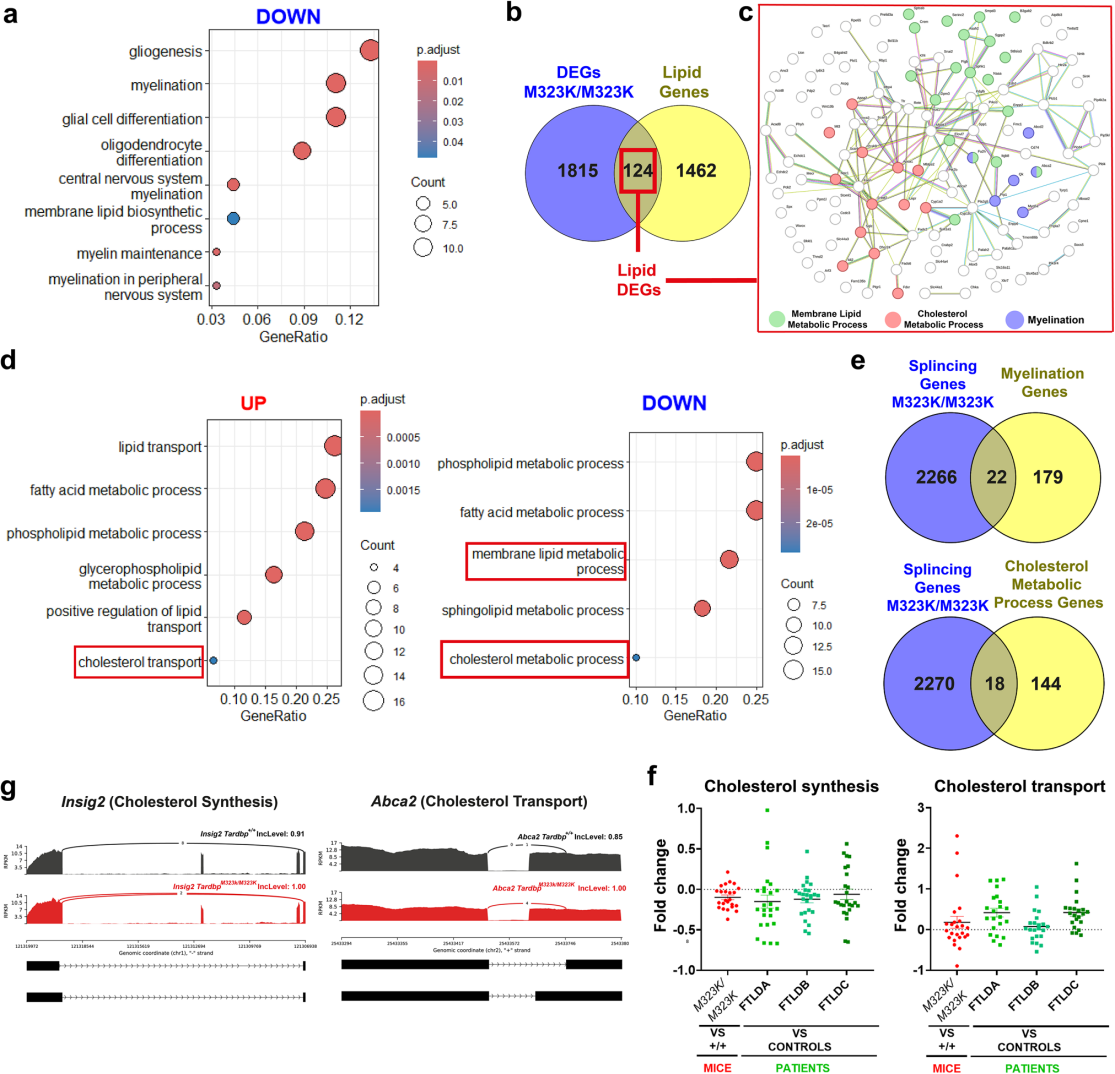
Transcriptomic and functional analysis of disrupted cholesterol metabolic processes in the frontal cortex of TDP-43 pathology. **a** Overrepresentation analysis (ORA) of the most significantly deregulated biological processes in the frontal cortex of 9-month-old male *Tardbp*^*M323K/M323K*^ mice, based on transcriptomic data. The size of the dots represents the number of genes involved in each pathway, while the color indicates the statistical significance level (adjusted *p *value). Experimental n per group: 4 *Tardbp*^+*/*+^ and 4 *Tardbp*^*M323K/M323K*^ male mice. **b** Venn diagram showing the overlap of differentially expressed genes (DEGs) in the frontal cortex with lipid-related genes. The lipid gene list was obtained by merging genes annotated under the Gene Ontology (GO) terms “lipid metabolic process” (GO:0006629) and “lipid transport” (GO:0006869). **c** Protein–protein interaction (PPI) network of differentially expressed lipid-related genes. A total of 120 lipid-related genes were identified and classified into 3 key biological processes based on GO terms: membrane lipid metabolism (green), cholesterol metabolism (red), and myelination (blue). **d** ORA of the most significantly deregulated biological processes, considering only the 124 lipid-related DEGs, analyzed according to their fold change. **e** Venn diagram depicting the overlap between genes with at least one splicing event (FDR < 0.05, Inc level > 0.5) and genes associated with myelination or cholesterol metabolism. **f** Sashimi plots illustrating alternative splicing events in *Dhcr7* and *Lpcat3*. **g** Comparative fold-change analysis of genes involved in cholesterol synthesis and transport between the frontal cortex of *Tardbp*^*M323K/M323K*^ mice and wild-type mice, as well as between FTLD Type A, B, or C patients and controls

To identify which genes and pathways were involved in the myelin and cholesterol homeostasis alterations identified, we performed a transcriptomic analysis by RNA sequencing of the frontal cortex from *Tardbp*^+*/*+^ and *Tardbp*^*M323K/M323K*^ 9-month-old male mice (*n* = 4 per group, using the other half frontal cortex to the one used for the lipidomics). A total of 226 differentially expressed genes (DEGs) using the threshold of p-adjusted < 0.05 were found in the cortex of the mutant mice (Supplementary Table 2). The Gene Ontology (GO) analysis by ORA (p-adjusted < 0.05 and fold change < 0) showed that the myelination process was significantly downregulated, further confirming our findings (Fig. 5a).

To elucidate how the alteration on TDP-43 led to dysregulation of lipid metabolism, and in particular, to the dysregulation of the endogenous cholesterol homeostasis, we looked at the consequence of the dysfunctionality of TDP-43 in RNA metabolism and splicing regulation of lipid regulatory genes. To specifically look for the lipid regulatory genes dysregulated in the mutant brains, we used a list of all genes that were annotated in the genome within the category of lipid metabolism (1586 lipid genes), as previously done [14] (Supplementary Table 3), to perform a targeted analysis within the total of 1939 DEGs (using threshold of *p* value < 0,05) (Fig. 5b). A total of 124 lipid genes (56 of them were targets of TDP-43) were deregulated (Supplementary Table 4), and after a STRING pathway analysis, those genes showed to be involved in membrane lipid metabolic process, cholesterol metabolic process, and myelination (Fig. 5c), further supporting our previous results. The GO analyses of those selected 124 lipid genes dysregulated in the mutant frontal cortex indicated an upregulation of cholesterol transport and a downregulation of cholesterol synthesis (Fig. 5d). We validated the gene expression level by quantitative PCR with a different set of biological samples using 9-month-old males and also 3-month-old females to discern the potential age and sex effect. In all of them, we confirmed the downregulation of regulatory genes of the cholesterol biosynthesis (*Srebf2, Dhcr24 and Sqle*) (Supplementary Fig. 4a), as well as the upregulation of some of the genes of the cholesterol transport (Supplementary Fig. 4b).

Since one of the main functions of TDP-43 is the regulation of RNA splicing, we investigated whether specific cholesterol regulatory genes, and also myelin regulatory genes, could also have modifications in their splicing as a consequence of the dysfunction of TDP-43. We performed a splicing analysis of the RNA sequencing data from the mutant and wild-type mice frontal cortex. A total of 2288 genes showed significant alterations in the different types of splicing events (SE, IR, A3SS, A5SS, MXE). From those, a total of 18 genes from the cholesterol metabolism regulatory pathways (12 of those were targets of TDP-43) had significant alterations in their splicing in the *Tardbp*^*M323K/M323K*^ cortex (Fig. 5e). We represented the significantly altered splicing exon skipping events by sashimi plots of one of the genes important for the cholesterol biosynthesis signaling pathway, *Dhcr7*, as well as of another gene related to cholesterol transport, *Lpcat3* (Fig. 5f). Interestingly, 22 genes related to the myelination process (14 of them were targets of TDP-43) had alterations in their splicing events induced in the mutant brain.

Finally, we looked at whether the downregulation of the cholesterol synthesis and upregulation of the cholesterol transport pathways were affected in the frontal cortex of patients with different TDP-43 proteinopathies. Thus, we selected all the genes involved in the cholesterol synthesis and transport pathways to see how they were affected in our mouse model of TDP-43 and compared those to other patients of TDP-43 proteinopathies transcriptomic databases. We re-analyzed a publicly available transcriptomic database study of the frontal cortex from different types of patients with frontotemporal lobar degeneration (FTLD) with TDP-43 pathology (type A, B, C and D) based on postmortem histological TDP-43 location and type of aggregates in different brain areas [35] Both the mice and the different types of FTLD-TDP patients showed the same trend of downregulated genes involved in the cholesterol synthesis and the upregulation of genes involved in cholesterol transport (Fig. 5h).

In conclusion, our findings suggest that TDP-43 alterations had an effect in the myelin stability and maintenance by directly downregulating the myelination process, but mostly through the downregulation of the biosynthesis of the endogenous cholesterol and upregulation of the cholesterol transport, altering the delicate balance of endogenous functional cholesterol, which is the essential component of the myelin. Moreover, the dysregulation of the cholesterol metabolism in TDP-43 proteinopathies can be manifested in the form of accumulated lipid droplets in the brain tissue, although further evidence is needed, including the visualization of lipid droplets in the brain by advance spectral techniques.

## Discussion

The primary objective of this study was to elucidate whether the myelin alterations observed in patients with neurodegenerative diseases associated with TDP-43 proteinopathy, as well as in mouse models carrying TDP-43 mutations, such as our TDP-43-M323K model, were at least in part a result of TDP-43 dysfunction that affected lipid metabolism. Specifically, we explored the causal relationship between TDP-43 alterations and the dysregulation of the endogenous brain cholesterol balance, since cholesterol is a main component of myelin and cell membranes and must be tightly regulated to ensure proper myelin formation and, consequently, the correct functioning of neurons.

In the frontal cortex of the TDP-43-M323K mouse model of TDP-43 proteinopathy, our lipidomic analysis revealed that the cholesterol metabolism was clearly altered in the mutant brains, pointing towards an accumulation of cholesterol esters and triglycerides in the form of lipid droplets, as well as a reduction in the levels of free cholesterol. Recent lipidomics analyses of the brain from FTLD-TDP patients reported a remarked loss of myelin-related lipids (sphigolipids) and accumulation of cholesterol esters in both frontal and parietal white matter, as a potential indicative of myelin loss [28, 38]. We confirmed the lipidomic findings of accumulated cholesterol esters in the form of LDs, in our mice and as well as in the brains of FTLD-TDP patients without a *TARDBP* mutation. The fact that these findings could be replicated in patients with TDP-43 pathology, but without a *TARDBP* mutation, supports the notion that the effects observed in these mice were not specific to this particular mutation, but rather reflected a more general TDP-43 dysfunction.

The endogenous accumulation of cholesterol esters has been suggested to be the consequence of the degeneration process, even the degeneration of the cellular membranes and the myelin. Here, we showed the impact of TDP-43 alteration on lipid droplet accumulation in a cell-autonomous manner in primary fibroblasts carrying the TDP-43-M323K mutation, further suggesting that the accumulation of LDs was also a direct consequence of TDP-43 dysfunction, rather than merely a result of a degenerative myelin or system process. The LD intracellular accumulation in TDP-43-related in vitro models of disease (for instance in iNeurons derived from iPSCs from *C9orf72* patients) [4, 26, 41] has been previously reported, further supporting the concept that the dysregulation of endogenous cholesterol is an early pathological event [18, 49], and not exclusively the consequence of a neurodegenerative process.

We further demonstrated that in the cerebellum of both FTLD-TDP patients [33] and TDP-43-M323K mutant mice—where no pathological TDP-43 signature was detected—PLIN2 immunoreactivity did not differ from that observed in control cerebella. These findings support the notion that TDP-43 pathology is associated with the alterations in PLIN2 expression.

Through the transcriptomic and splicing analyses, we found that the main molecular mechanism impacting the cholesterol disruption observed was through the dysregulation of the endogenous cholesterol metabolism pathways. The regulation of the brain cholesterol, in particular the endogenous cholesterol biosynthesis, has been previously shown to be downregulated in several neurodegenerative disorders [46], even in the physiological process of aging [47, 50]. For instance, we have previously reported similar transcriptomic alterations of the cholesterol biosynthesis in the spinal cord of SOD1^G93A^ mice at early pre-symptomatic disease stages [14]. Other groups have also described very early cholesterol alterations in other models of TDP-43 mutations [12, 48]. Thus, a reduction in the endogenous cholesterol biosynthesis seems to be one of the most important and shared molecular mechanisms from early stages of the neurodegenerative processes. This is crucial, as many neurodegenerative disorders share common pathological mechanisms.

In this study, we demonstrated that the FTLD-TDP postmortem frontal cortices exhibited a more pronounced downregulation of cholesterol biosynthesis genes compared to both the control aged donors and the mutant mice. This could be attributed to the fact that the mice, at that particular age, did not exhibit significant neurodegeneration. The FTLD-TDP brains also show increased lipid droplet accumulation compared to aged controls, as demonstrated by the staining results. On the other hand, alterations in cholesterol transport genes were not consistently upregulated across all FTLD-TDP patient subtypes, as observed in the FTLD-TDP type B group, which included a higher number of ALS patients, potentially with less frontal cortex damage than the other FTLD-TDP types. This was also observed in the mice, which further suggested that more advanced neurodegeneration in the frontal cortex is associated with a broader dysregulation of cholesterol metabolism, with cholesterol biosynthesis being one of the earliest changes observed in all cases. In addition, these mutant mice exhibited myelin alterations from an early age [17], supporting the idea that cholesterol dysregulation, which impacts myelination, may be an early event triggered by TDP-43 alterations.

Finally, we looked closer at the specific expression and splicing of the TDP-43 target genes to evidence the role of the TDP-43 mutation dysfunction, or at least contributing, to the cholesterol and myelination alterations observed in this study. Other studies have suggested a direct role of TDP-43 dysfunction in lipid regulation alteration and obesity, inferring a general role of TDP-43 in the body lipid metabolism [8, 48].

### Closing remarks and future perspectives

This study highlights the critical role of cholesterol dysregulation, particularly in endogenous cholesterol biosynthesis, as a common event in neurodegenerative diseases associated with TDP-43 pathology. The findings suggest that altered cholesterol metabolism, which impacts myelination, could serve as an early indicator of TDP-43 dysfunction, opening new avenues for early diagnostic strategies. Further studies are needed to disentangle the relationship between TDP-43 dysfunction and lipid metabolism. Understanding the broader implications of cholesterol dysregulation in neurodegeneration may offer novel therapeutic targets to address both the underlying pathology and the progression of these diseases, ultimately advancing clinical approaches to treatment.

## Supplementary Information

Below is the link to the electronic supplementary material.

Supplementary file1 (XLSX 39 KB)

Supplementary file2 (PDF 311 KB)

## Data Availability

Data are provided within the manuscript or supplementary information files. Sequence data that support the findings of this study are deposited in GEO openly accessible: GSE294236.
