# Supplementary material for: TDP-43 dysregulation impairs cholesterol metabolism linked with myelination defects
Source: Acta Neuropathol. 2025 Sep 4;150(1):23. doi: 10.1007/s00401-025-02927-x (PMC12411602; doi:10.1007/s00401-025-02927-x)
Supplement: Supplementary file 2 — Supplementary file2 (PDF 311 KB) [file 401_2025_2927_MOESM2_ESM.pdf]

Supplementary Fig.

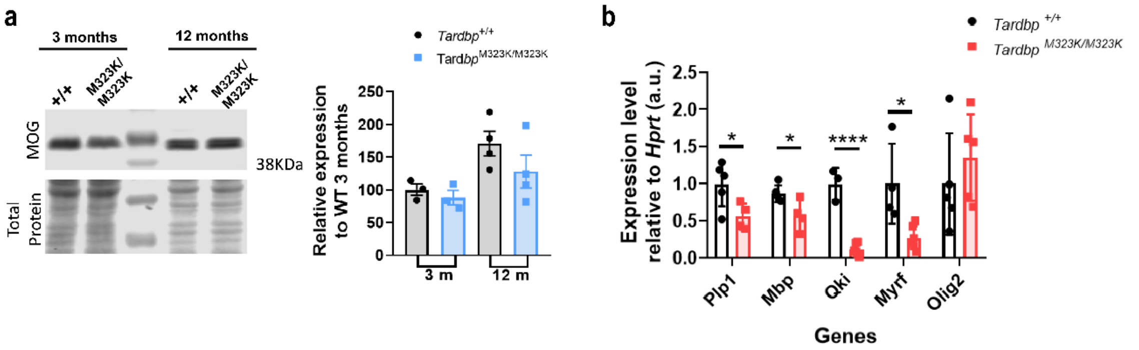

Supplementary Fig. 1. (a) Western blot analysis of MOG protein levels in frontal cortex samples at 3 and 12 months of age, with quantification relative to wild-type (WT) at 3 months. **(b)** Relative expression of myelin- and oligodendrocyte-related genes (*Plp1*, *Mbp*, *Qki*, *Myrf*, *Olig2*) normalized to *Hprt* in frontal cortex samples from $Tardbp^{+/+}$ and $Tardbp^{M323K/M323K}$ mice. Experimental N per group: 3-5 $Tardbp^{+/+}$ and 3-5 $Tardbp^{M323K/M323K}$. Data are presented as mean ± SEM. *p < 0.05, ****p < 0.0001.

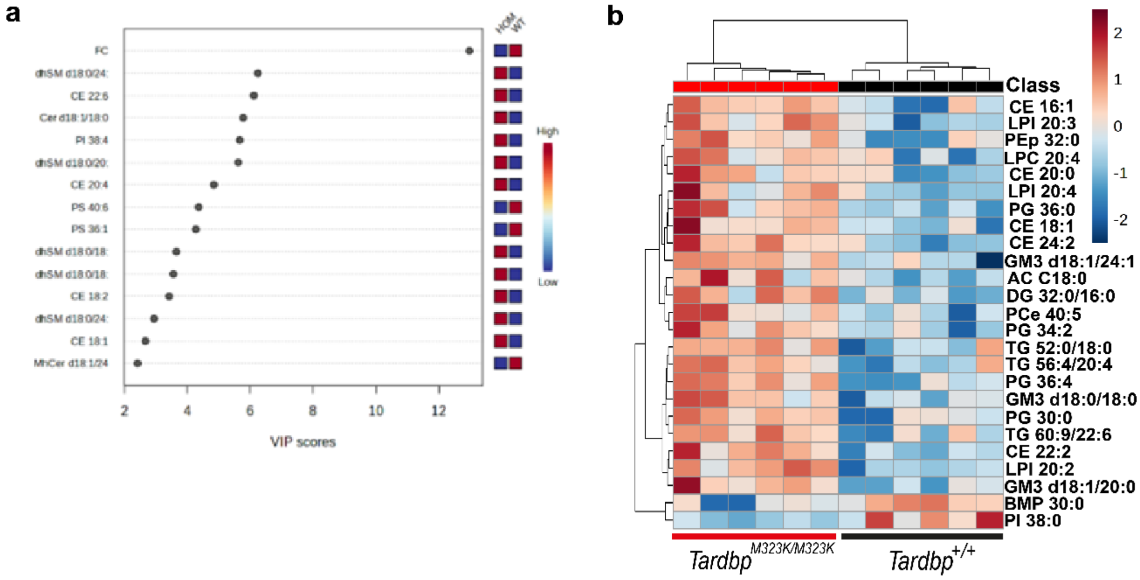

Supplementary Fig. 2. (a) Vip score **(b)** Heatmap representation of hierarchical clustering of individual samples according to the top 25 statistically significantly different lipid species.

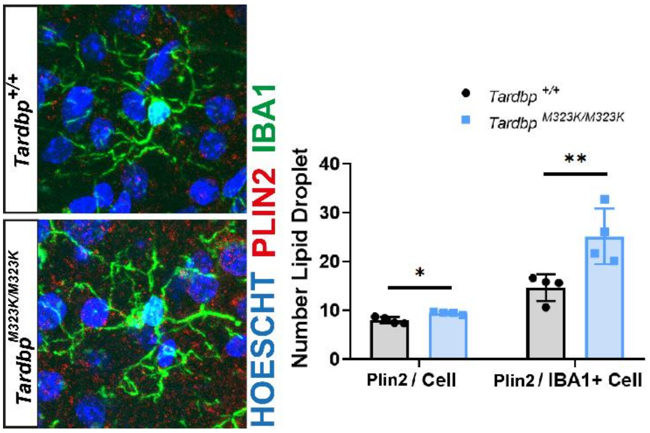

Supplementary Fig. 3. Increased microglial PLIN2 accumulation in *Tardbp*$^{M323K/M323K}$ mouse frontal cortex. Representative confocal images of cortical sections from *Tardbp*$^{+/+}$ and *Tardbp*$^{M323K/M323K}$ mice stained for IBA1 (green, microglia), PLIN2 (red, lipid protein), and Hoechst (blue, nuclei). Quantification of PLIN2 per cell and per IBA1+ cell reveals a significant increase in the number of PLIN2 in mutant mice compared to wild-type controls ($n = 4$ per group). Data are presented as mean ± SEM. $P < 0.05$, $P < 0.01$, unpaired t-test. Scale bar: 10 µm.

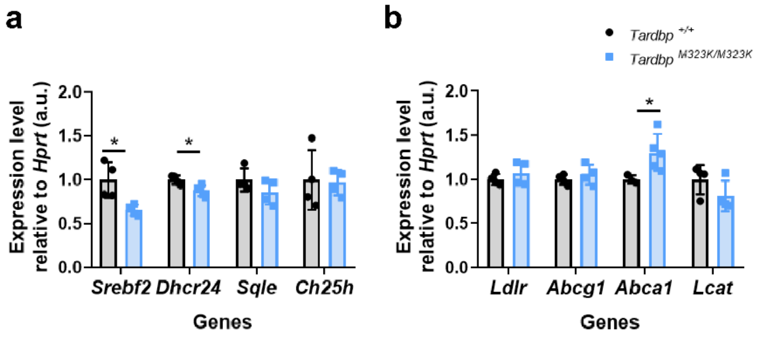

Supplementary Fig. 4. (a) Relative expression of cholesterol synthesis (*Srebf2*, *Dhcr24*, *Sqle*, *Ch25h*) and **(b)** cholesterol transport (*Ldlr*, *Abcg1*, *Abca1*, *Lcat*) normalized to *Hprt* in frontal cortex samples from *Tardbp*$^{+/+}$ and *Tardbp*$^{M323K/M323K}$ mice. Experimental N per group: 4-5 *Tardbp*$^{+/+}$ and 3-5 *Tardbp*$^{M323K/M323K}$. Data are presented as mean ± SEM. *$p < 0.05$.
